# Creating a Community Coalition to Prevent Childhood Obesity in Yakima County, Washington: Rev It Up! 2008

**DOI:** 10.5888/pcd9.110243

**Published:** 2012-07-05

**Authors:** Robin Johannsen Faubion, Jessica Brown, Ruth C. Bindler, Kris Miller

**Affiliations:** Author Affiliations: Jessica Brown, Yakima Public Health District, Union Gap, Washington; Ruth C. Bindler, Kris Miller, Washington State University College of Nursing, Spokane, Washington.

## Abstract

**Background:**

One-third of the US population is obese, and childhood obesity has tripled since the late 1970s. Childhood obesity is a significant health issue requiring interventions on individual, interpersonal, community, organizational, and policy levels. Community coalitions offer successful strategies for engaging community partners with health improvement goals.

**Community Context:**

In 2008, Yakima County, an agricultural community in eastern Washington, was ranked the eighth fattest city in the United States. Recognizing the obesity problem, the Yakima Health District (YHD) established 2 objectives: to decrease rates of childhood obesity in Yakima County and to recruit and establish a community coalition of key stakeholders and experts to help address the problem.

**Methods:**

The YHD spearheaded a movement to create a community coalition. The coalition applied for and received state and federal grants. In September 2008, the YHD held the first recruitment event for Rev It Up!, its community-based effort to address the obesity problem in Yakima. YHD invited the Washington State Department of Health to advise the coalition-building and action-planning process.

**Outcome:**

The community coalition achieved 5 of 7 objectives, including developing a common vision, creating an advisory committee, and conducting a community inventory, prioritization process, and action plan. However, unexpected public health challenges in the YHD delayed coalition efforts.

**Interpretation:**

Creating the Rev It Up! coalition met a community need and engaged community partners. Some potential partners were dissuaded by the 6-month period required to establish the coalition. Rev It Up! continues as a community effort to reduce rates of obesity in Yakima County.

## Background

One-third of the U.S. population is obese, and the rate of childhood obesity has tripled since the late 1970s. Although obesity rates have stabilized in the past 2 years, they are not on the decline (1,2). Obesity is defined in adults as a body mass index (BMI) of 30 kg/m^2^ or higher and in youth as a BMI-for-age percentile of 95% or higher (3). Obesity in adults and children results when energy consumption exceeds energy expenditure. Many genetic, behavioral, environmental, social, and cultural factors contribute to excess energy consumption. Obesity is a significant health issue and increases the risk for cardiovascular disease, type 2 diabetes, hypertension, stroke, and certain types of cancer, including endometrial, breast, and colon cancer (4). The best predictor of adult obesity is pediatric obesity (5,6). Thus, early intervention is essential to obesity prevention.

Successful intervention requires a multilevel approach. The socioecologic model of health promotion (7) targets individual, interpersonal, community, organizational, and policy-level changes. The model provides a theory base for community prevention efforts (8) and targets the many factors contributing to excess energy consumption. Multiple levels of intervention require a concerted effort by a group of individuals with diverse skills.

Community coalitions, composed of a diverse group of community members who are committed to effecting change, are successful in engaging community partners in health improvement (9,10). The collaborative work of a coalition is more effective than the efforts of individuals or individual groups, because it is composed of partners representing multiple sectors, reduce duplication of effort, and use various resources to accomplish a common goal (11).

## Community Context

Yakima County is an agricultural community in eastern Washington with a population of 243,231 (12). It is considered one of the best apple-growing regions in the world, and it produces 75% of the nation’s hops. Yakima farmers also grow cherries, pears, peaches, and grapes, among other commodities. Yakima averages 300 days of sunshine, 9 inches of precipitation, and 195 growing days per year (http://www.yakima.org/yakima/index.html).

Despite its agricultural bounty and number of sunny days, Yakima earned the ranking of the eighth-fattest city in America in 2008 (13). According to the Washington State Department of Health (WA State DOH), 29.7% of the population of Yakima are obese (BMI ≥30 kg/m^2^), and 35.6% are overweight (BMI, ≥25 kg/m^2^) (14). In Yakima, 22% of the population have an income below the poverty level, and the median household income in 2009 was $41,055 (12).

Hispanics make up 45% of the population of Yakima County. The Yakima Indian Reservation, located in the lower Yakima Valley, comprises 4.3% of the county’s population, and whites and other non-Hispanics, along with a small representation of other ethnic minority groups, make up the remainder (12).

Childhood and adult obesity is a significant issue in Yakima County. WA State DOH provides information on youth obesity and physical activity through the Healthy Youth Survey database (15). The Healthy Youth Survey, which was first implemented in 1988, is modeled on multiple nationwide surveys, including the Behavioral Risk Factor Surveillance Survey (BRFSS). Health care workers, coalitions, and public health practitioners use the survey to guide policies and programs designed for youth. In 2006, 27.8% of Yakima County eighth- and tenth-graders were overweight, and 31.9% were obese. In 2008, these percentages increased to 30.2% overweight and 35.4% obese. The Healthy Youth Survey shows an increase in the amount of screen time and a decrease in physical activity during the same 2006 to 2008 period.

The Yakima Valley Farm Workers Clinic is a local community health clinic for Yakima’s underserved population. The clinic has 6 locations and in 2006 served 13,815 patients aged 2 to 19 years. Of these patients, 26.4% were obese (Yakima Valley Farm Workers Clinic, unpublished data, November 2007).

The Yakima Health District (YHD) provides education and health care services to promote health and prevent disease. YHD was founded in 1911, and its successful intervention in a typhoid outbreak that year was the impetus for establishing county health districts throughout the country (16). Recognizing the current trends of obesity in Yakima County, YHD established 2 main objectives: to decrease obesity in Yakima County and to recruit and establish a community coalition of key stakeholders and experts to help address the problem. To achieve these objectives, YHD outlined 4 general strategies:

1. Coordinate countywide efforts on childhood obesity prevention (later expanded to an effort to prevent obesity in the overall population)
2. Secure funding to sustain, expand, or implement programs to prevent childhood obesity in Yakima County
3. Act as a community resource for residents, educators, and professionals to provide accurate information on and tools for addressing childhood obesity
4. Advocate for policies that promote healthy lifestyles in Yakima County

The community coalition identified the following goals:

1. Develop a common vision
2. Form an advisory committee
3. Review data; determine and conduct assessments as part of a community inventory
4. Develop coalition criteria and complete a prioritization process
5. Develop an action plan for prioritized recommendations, including action steps, using the framework of the Washington State Nutrition and Physical Activity Plan
6. Determine strategies for implementing the action plan
7. Begin implementation of the action plan

## Methods

Several community agencies had been involved in obesity prevention and treatment before the Yakima County community coalition was established, but there had been little collaboration and no central leadership. Agencies applied to the same local foundations for funding for similar efforts. Obesity overall was recognized as a local health issue, but no community effort on obesity prevention existed. In 2006, the YHD conducted a community needs assessment using the Mobilizing for Action Through Planning and Partnership (MAPP) tool (9). The assessment had 2 objectives: to determine the community’s awareness of and concern about obesity and to discover which population groups might benefit most from intervention. The assessment showed that community leaders considered obesity to be the most important health issue in Yakima County. Community leaders also indicated that obesity was not being adequately addressed by public health partners. Population groups most likely to benefit included school-aged children, especially members of certain racial/ethnic minority groups (Hispanic and American Indian) and those living at less than 185% of the poverty level.

As a result of the community needs assessment, YHD appointed a community assessment specialist in September 2007 to spearhead the obesity prevention effort. Representatives from community agencies who were already directly or indirectly involved with obesity prevention and treatment were invited to meet and discuss how to coordinate efforts. The 7 agencies invited via personal contact (telephone call or e-mail) included 3 local health care facilities (Yakima Valley Memorial Hospital, Yakima Pediatrics Associates, and Yakima Valley Farm Workers Clinic), the local educational services district (ESD 105), and 3 community service agencies (North)were usedwest Community Action Center, YMCA, and 21st Century Community Learning Centers). Representatives from these agencies committed to YHD’s goals and objectives for intervention and community engagement; they decided on a name, Rev It Up!, with Yakima County. Thus, the first strategy, coordinating countywide efforts, had been successful.

The real work of creating a community coalition would occur during the next 24 months. YHD applied for and received both federal and state grants between October 2007 and September 2008. Funding from a WA State DOH Preventive Health and Health Services (PHHS) block grant and from the Basic Food and Nutrition Education Program (BFNEP) facilitated the initiation of 2 programs aimed at school health. Part of the PHHS block grant funded Rev It Up! with Healthy Choices, a program designed to help school districts enhance, revise, implement, and evaluate wellness, nutrition, and physical activity policies. These policies were required for compliance with Washington State Substitute Senate Bill 5436 (17). Many district policies were vague and did not include implementation strategies. Rev It Up! with Healthy Choices provided eight $2,000 scholarships to help school districts review and revise their policies and gave the districts access to YHD staff knowledgeable on policy development.

The BFNEP grant helped to fund Rev It Up! with CATCH, an after-school program that used the Coordinated Approach to Child Health (CATCH) curriculum, which emphasizes nutrition and physical activity (18). Designed for school-aged children, the CATCH program directly addressed 4 of 8 school health components set forth by the Centers for Disease Control and Prevention: health education, physical education, nutritional services, and family/community involvement.

With implementation of the initial program objectives under way, the next step was to engage the community and recruit a group that would develop an action plan and introduce the Rev It Up! coalition to the entire community. On September 17, 2008, YDH and its partners held the first Rev It Up! recruitment event. Of 102 people invited, including teachers, school district representatives, school nurses, health care workers, parks and recreation managers, nursing school students and faculty, community activists, and interested community members, 59 attended. A prominent Yakima pediatrician with a passion for childhood obesity prevention presented a keynote address. The audience voted on a logo for Rev It Up! and volunteered for various positions. The Hispanic population was well represented, especially by physical education teachers from lower Yakima Valley. As a result of the recruitment event, 57 people indicated an interest in becoming involved in the Rev It Up! coalition. The number of community agencies represented in the coalition increased from 8 to 15. Joining the coalition were representatives from the city of Yakima, Yakima Athletic Club (YAC), YAC Fitness, 4H, the Yakima Greenway Foundation, Fit Kids USA, and the Washington State University College of Nursing. An advisory committee was formed to provide leadership for the growing coalition. Advisory committee members were individual volunteers and volunteers representing community agencies.

With the new momentum and increased number of volunteers, Rev It Up! leadership invited the WA State DOH to advise the coalition-building and action-planning process. During the action-planning process, the coalition amended its strategies to include adult obesity prevention. The Washington State Nutrition and Physical Activity Plan, developed by the WA State DOH, and 2 Healthy Communities tool kits (www.cdc.gov/healthycommunitiesprogram/) were used to help communities promote health and to assist local policy makers in improving community environments (19–21). The first advisory committee meeting took place in December 2008; during the meeting, a timeline for developing an action plan was introduced and commitment from members was confirmed. The advisory committee met monthly during the first quarter of 2009, and subcommittees were established to conduct a community inventory. The purpose of the inventory was to identify strengths and barriers to improving nutrition and increasing physical activity. Subcommittee members and community volunteers conducted the inventory using questionnaires from the Healthy Communities Tool Kit 1 (20). Local high-school students were recruited to inventory grocery stores, convenience stores, and eating establishments. The county geographic information services provided maps of all local eating establishments. A walkability/bikeability subcommittee evaluated the ease of walking and biking throughout Yakima County, and another subcommittee examined the accessibility of recreational facilities. One committee member gathered data on school food service menus and provided sample menus with nutritional information. The advisory committee was surprised to learn, for example, that elementary-school students were regularly offered doughnuts and Pop-Tarts for breakfast.

By March 2009, the inventories were completed and reports were provided to advisory committee members. The advisory committee selected priority recommendations and decided on 4 areas of focus for the coalition: 1) improve school wellness, 2) promote active community environments, 3) reduce food insecurity, and 4) increase the proportion of mothers who breastfeed (Table). Actions steps were included for each area of focus.

**Table:** Action Plan Priority Recommendations

Action Plan Priorities	Action Steps
**Priority no. 1**	
Increase access to healthy foods and increase physical activity in children; create school wellness policies.	Implement school wellness policies — vending programs, food service, farm to school, school gardens
	Open gyms
	Increase physical activity during school day — competitive and collaborative activities
	Provide more active recess
	Open schools for family activities
	Replace revenue from vending machines with fundraisers, etc
	Create farm-to-school workshop
**Priority no. 2**	
Provide active community environments. Create city comprehensive plans.	Work with cities, county, organizations, and businesses to influence changes in communities
	Increase green space, open fields
	Provide connectivity to neighborhoods
	Improve transportation
	Develop trails and sidewalks
	Create bike lanes
	Create pocket parks in neighborhoods
	Require a park per number of residential homes
	Provide adequate lighting, public restrooms, and water
	Locate a central commercial area within walking distance of residential area
	Allocate community funding to provide needed changes
**Priority no. 3**	
Reduce food insecurity. Work with food banks and Basic Food program.	Recruit volunteers for food banks and train them in Basic Food practices
	Improve access to healthy foods at food banks
	Educate about food preparation and food dollars
**Priority no. 4**	
Increase breastfeeding.	Promote breastfeeding, in doctor’s offices and in prenatal care facilities
	Provide breastfeeding education
	Create breastfeeding-friendly workplaces

During the development of the action plan, YHD experts were developing the Rev It Up! media campaign and website with the help of volunteers and agencies. The media campaign aimed to inform the community about childhood obesity, increase awareness of the issue, attract volunteers, and create community advocates. Rev It Up! sponsored 6 months of television advertisements, which aired in the first half of 2009, emphasizing the importance of physical activity and nutrition.

**Rev It Up! television advertisement (WMV, 6Mb):** Announcer It’s no surprise. Americans are getting heavier and Yakima County is no exception
Announcer with over 60% of the county population either overweight or obese.
Announcer The Rev It Up! coalition is working to help kids
Announcer and families eat healthier and be more active.
Announcer You can help
Announcer limit television and video time to no more than 2 hours per day.
Announcer Be active with your kids and encourage kids to be
Announcer physically active at least 1 hour a day every day.
Announcer Try to eat at least 2 more fruits and veggies every day.
Announcer The more fruits and veggies you eat, the better.
Announcer To find out more about the Rev It Up! coalition, visit www.RevItUpYakima.org

Rev It Up! promotional items (pedometers, key chains, pens, and MyPyramid magnets) were distributed at community events. The Rev It Up! website was developed as an active resource for community members, health care providers, educators, and parents on obesity prevention (www.revitupyakima.org).

In September 2009, the final events for the advisory committee timeline — community input meetings and a community kick-off, scheduled for late summer and early fall 2009 — were put on hold because of other public health challenges in Yakima County, including the H1N1 influenza emergency.

## Outcome

A community coalition was created, and it met 5 of its 7 goals: it developed a common vision, formed an advisory committee, conducted assessments as part of a community inventory, completed a prioritization process, and developed an action plan. Advisory subcommittees met regularly through August 2009 to focus on school wellness and breastfeeding promotion. The unexpected public health challenges presented by H1N1 influenza assumed priority, and the action plan was put on hold.

During this delay, some community partners lost motivation (10 individuals withdrew entirely). Many, however, remained committed to the common vision. The Rev It Up! coordinator continues to communicate with 57 community partners via the Rev It Up! e-mail database. The e-mail database serves as a tool to schedule meetings and disseminate information among committee members.

## Interpretation

Creating the Rev It Up! coalition was worthwhile. The need in Yakima County was apparent, and community leaders, partners, and individuals participated. We began recruiting community partners and implementing projects before developing an action plan, and if we were to do this again, we would have a clear plan of action before recruitment that included goals, objectives, and outcomes for the coalition. We may have lost some potential partners during the 6-month period of establishing the coalition by not having an action plan. When we realized the need for planning, we requested and received assistance from the WA State DOH.

Recommendations to others would include encouraging public health practitioners to develop a database and use electronic communication to inform partners. Electronic communication allowed our coalition to remain intact despite the H1N1 outbreak and subsequent interruptions.

We also discovered that people tire of the planning process. Many are willing to work on projects, but the coalition-building effort required more commitment than many were willing to make. Today, as we refocus on our priority areas, we are striving to keep the planning process at the advisory level and recruit community volunteers only to participate in actual projects. Our perception is that people are ready to work and but tire of planning.

Overall, our experience was positive, and the process continues to evolve. The Rev It Up! Coalition is an ongoing community effort to reduce obesity in Yakima County. What began as a focused effort on childhood obesity has become a more comprehensive effort aimed at reducing obesity in the entire community. Many projects remain focused on youth, but others include adults. Obesity affects the health of the entire community, and persistent effort is needed to effect change.
